# Dr. Brown's Case of Constipation

**Published:** 1809-06

**Authors:** William Cullen Brown

**Affiliations:** Surgeon of H. M. S. Woolwich


### *To the Editors of the Medical and Physical Journal.*

Gentlemen,

James Levenny, ætat. 24, ordinary seaman, of a well defined melancholic temperament; lately belonging to his Majesty's ship Ocean. From his infancy he has laboured under a singularly costive habit of body, which has been a constant source of uneasiness and distress to him. He has been generally accustomed to void the contents of the intestines once in five or six days, but has been repeatedly from eighteen to twenty-five days without a passage. He has been twelve years at sea; during all this time, particularly latterly, has experienced bad health, having been for the most part in the surgeon's list; for these last two years, he has been entirely incapable of doing his duty, in consequence of his infirmity having increased to an almost incredible degree.

The symptoms under which he laboured, previously to the last two years, were occasional sickness at the stomach; pain in the chest, about the sternum, aggravated by a short short dry cough; loss of appetite; prostration of strength; flatulency, and dejection of spirits. About two years ago, the above complaints were greatly aggravated by the supervention of frequent vomiting, in consequence of which almost every thing taken by the mouth was quickly rejected. The returns of constipation have since become much more obstinate and frequent, attended with severe pain of his bowels, and greater general uneasiness.

About nine years ago, when employed in the service of one of the Berwick Companies, he was under the necessity of applying for relief to the Royal Infirmary of Edinburgh, where he continued as long as eight months, under the care of the ordinary physician of that hospital, Dr. James Hamilton; the mention of whom implies that every thing must have been done for him that could be devised by a practitioner of experience and judgment. According to the man's account, neither the purgatives then given him by the mouth, nor the clysters thrown up, any more than various external applications, were productive of benefit; the only remedy which afforded him relief, and that not always, and even when it did, but temporarily, having been the employment of the hot bath, which, however, always left him for several days in a state of great debility. At the expiration of the eight months, he was removed from the infirmary, by the master of the smack to which he belonged, little, if at all, relieved at the time he left it, his stools never being passed but after an interval of seven or eight days. Immediately on his arrival in London, he was conveyed to St. Thomas's Hospital, in which he continued ten days without deriving any advantage from the remedies employed.

Latterly, while he continued on board of Lord Collingwood's ship, the Ocean, Dr. Gray, Physician to the Mediterranean Fleet, and Mr. Fullarton, Surgeon of the Flag ship, tried every remedy in their power, that had ever been suggested in similar cases, but with no better success than had attended the exertions of Dr. Hamilton, about seven years before, every thing taken by the mouth being almost immediately returned, and all manner of injections as instantaneously coming off unchanged.

At the time of his being under the care of Dr. Gray and Mr. Fullarton, he was attacked with a torpor of the lower extremities, amounting to a complete paraplegia, which continued several days, and was at length with difficulty overcome, by means of friction, with warm flannel and stimulant applications.

From

From this time his complaints becoming daily worse, and his costiveness more obstinate, on the 13th of November, 1808, he was sent to the Naval Hospital at Malta, where he continued without relief, (being only five days in the Hospital) until the 26th of the same month, when he was invalided, and sent for a passage to England in this ship. The remedies employed by Mr. Allen, Surgeon of the hospital, were similar to what had already been had recourse to.

The day after his arrival on board of the Woolwich, (November 27th 1808,) he had a stool for the first time for twenty-seven days, from the time he had had one on board of the Ocean. From this time to December 29th following, (including thirty-two days,) he remained without another motion of his bowels, notwithstanding a variety of remedies tried, either in succession or combination. The account, indeed, that the man gives is discouraging to the urging of means of relief; as he uniformly affirms that, on all former occasions of such paroxysms of constipation, the remedies used had no decided effect in relieving him, a stool generally taking place in consequence of the action of the intestines being somehow roused, when least expected, and the paroxysm usually running its course, as it were, and at length yielding spontaneously. Notwithstanding this unfavourable account, between November 27th, and December the 29th, every endeavour was used to procure an evacuation of his bowels. His extreme debility forbade bleeding. Magnesia vitriolata in divided doses, rhubarb combined with calomel, bitter extract, jalap, and other purgatives, were employed ineffectually, being quickly thrown up again. Injections of salt and water, of natron vitriolatum, of Castor oil, and various others, had no better effect. His abdomen was rubbed with tepid oil, and he was afterwards made to sit with a blanket wrapped round him, over a tub of hot water, to as little purpose. In the mean time his strength sinking daily, for want of nourishment, port wine, porter, sago, and soups were given him, though generally thrown up in a few minutes. Thirty-two days passed in the repeated employment of these, and similar remedies, to no purpose; when at length he felt a call to evacuate, which did not seem to be imputable to any thing that had recently been resorted to. Unfortunately the fæces were thrown away uninspected.

From December the 29th, 1808, to the present period (March 17th, 1809) making seventy-eight full days, he has been been without an alvine evacuation. The means already spoken of, have been again and again repeated and varied according to circumstances, and semicupium has been employed in vain. He is now in the last stage of emaciation, and his strength is reduced to such a degree that he is incapable of raising himself in his hammock without assistance. His only support has been the little that has been absorbed of the port wine, or soup, previously to its being thrown up after being received into the stomach.—An infusion of tobacco has been lately three times injected at proper intervals, and returned in a couple of minutes unchanged. Tobacco smoke has likewise been introduced as often, without any other effect than once occasioning a great discharge of flatus, both upwards and downwards, attended with considerable relief to the pain in his bowels and his general uneasiness. A suppository, composed of rhubarb and jalap, moulded into a proper shape by the addition of mucilage, the day before yesterday was introduced up the rectum, with the probability of producing some good effect by more permanently stimulating the gut; but no other result than much uneasiness to the poor man has taken place. It is now proposed to immerse him in a general hot bath of sea water in the quarter gallery of the ship, and to detain him in it as long as circumstances will admit.

March 18th. He was kept in the bath yesterday for twenty minutes at the heat of the blood, and expressed much satisfaction at its general good effect in soothing his uneasy feelings. Towards this morning, after a violent exertion, he passed an indurated ball of fæces of considerable size, accompanied with the discharge of a quantity of blood, from which he experienced some relief. To day at noon he was again immersed in the bath at the temperature of 100° of Farenheit's thermometer, where he continued only about twelve minutes, when he was threatened with a syncope, from which, however, he was roused by first one and shortly after another glass of Madeira. Immediately after being taken out of the bath and wiped dry, a strong desire for a stool came on, and with much pain and difficulty he passed four large hard lumps, as far as he could judge by his sensations, similar to the one that had come from him the preceding day. The whole of this afternoon he has felt himself much easier, but has had a great inclination for another stool, in attempting to procure which, while supported on the bed-pan, he fell into a faint, a faint, and was recovered from it by the employment of diffusible stimuli and external means.

March 19th. He has had several fainting fits in the course of the day, but still no stool. Hysteria seems to predominate greatly on occasion of these fits, which always end in a flood of tears. Tea, soft bread and butter, and a bit of fowl have been taken by him, and have remained longer on his stomach than usual; still his strength appears to be sinking.

March 20th. All of this day has been nearly as yesterday, with the exception of both the upper and lower extremities having become paralysed. Food, which he had taken twenty-four hours before, was this morning thrown up little changed. Friction with warm flannel has been employed to his arms and legs with some benefit. To day he has shewn a particular vacancy of look, and has spoken a great deal incoherently; though he can be quickly made to rally his ideas, and resume his usual manner. He has had a little nourishment forced upon him, and has been occasionally supported with wine, diluted spirits, and tincture of opium.

March 21st. The torpor of the extremities and other symptoms continue much the same. Has been treated on a similar plan as hitherto. No stool yet.

March 22d. His fainting fits are now more frequent, and of greater duration. He has taken tea and a little toast and butter for breakfast, and some roast mutton for dinner, with some return of appetite. No stool.

March 23d. Has talked incoherently the greater part of the day, and been attacked thrice with fainting, but he takes a little food without immediately throwing up. The sensation in his extremities is now a good deal recovered. To go on as hitherto.

March 24th. This morning at six o'clock, he had a stool consisting of ten or twelve scybala, each about the size of a walnut. It is judged that he is since much easier, with respect to the pain in his bowels, from the cessation of a peculiarly plaintive moaning with which his sufferings for many days past have been accompanied, his own report not being to be depended on from the wandering of his mind. He is not conscious of having had a stool, and is become fractious with all his attendants, except his medical. He now begins to move his legs, and calls much for grog and meat, the latter of which, when presented to him, he only nibbles. He has had one hysterical paroxysm this day. He is in future to be allowed wine instead of of spirits, by way of cordial; for breakfast tea and toast, and for dinner something nourishing and of easy digestion. The friction of his extremities with flannel to be continued, and night attendants to watch over him.

March 25th. Last night, about twelve o'clock, he had another stool of similar consistency, but not so copious as the preceding. The whole of this day he has talked quite incoherently, but taken food somewhat better, and remained easy.

March 26th. The same delirious manner has continued all this day. He has had no stool, but taken some nourishment.

March 27th. His intellect this morning is quite clear, and he says that he is unconscious of what has passed these three days. The paralysis of his extremities now almost removed, and his appetite is returning. He feels nothing but extreme debility, and things now mostly remain on his stomach.

April 18th. Since last report he has mended hourly; his appetite is greatly improved, as well as his strength, and he has had a natural stool every six or seven days, which in his case may be considered as no more extraordinary than other people having one once in twenty-four hours.

The obvious inference to be drawn from the case is, that no patient in similar circumstances ought to be deserted as incurable, and that patience and perseverance in medical practice, as in most other cases, will generally be rewarded with success.

I am, &c.

WILLIAM CULLEN BROWN, M. D.

*May* 18, 1809. Surgeon of H. M. S. Woolwich.

